# Substrate dependent growth conditions and moisture dynamics drive aroma development in solid-state *Aspergillus oryzae* (koji) fermentations of cereal and oilseed processing side streams

**DOI:** 10.3389/fmicb.2026.1829344

**Published:** 2026-05-21

**Authors:** Sylvester Holt, Fiona Melzer, Cristiana Paola Persico Pivcevic, Karsten Olsen, Dennis Sandris Nielsen, Lorenzo Tirelli

**Affiliations:** 1 Section for Design and Consumer Behavior, Department of Food Science, University of Copenhagen, Frederiksberg C, Denmark; 2 Section for Food Microbiology, Department of Food Science, University of Copenhagen, Gut Health, and Fermentation, Frederiksberg C, Denmark; 3 REDUCED Aps, Frederiksberg C, Denmark

**Keywords:** aroma, cereal, food side-streams, GC-MS, koji, oilseed, solid-state fermentation

## Abstract

**Introduction:**

Solid-state fermentation (SSF) with the filamentous fungus *Aspergillus oryzae* is widely used in traditional fermentations, yet the mechanisms governing aroma formation on agro-industrial side-streams remain poorly understood. This study investigated how substrate composition and dynamic moisture conditions influence physicochemical changes, metabolic activity, and volatile organic compound (VOC) formation during SSF.

**Methods:**

Wheat bran, rye bran, rapeseed press cake, and pumpkin seed press cake were fermented with *Aspergillus oryzae* under varying moisture conditions. Fermentation progression was monitored through measurements of fungal growth, pH, water activity, reducing sugars, and primary amines. VOC profiles were analyzed over time to evaluate substrate-specific and moisture-dependent aroma development trajectories.

**Results:**

Fermentation behavior differed markedly between substrates and moisture conditions. Wheat and rye bran followed similar trajectories characterized by decreasing pH, moderate moisture loss, and increased reducing sugars and primary amines. Rapeseed press cake showed alkalinization and strong amine accumulation, whereas pumpkin seed press cake displayed limited pH change but pronounced decreases in water activity and proteolytic signatures. VOC analysis revealed a directional transition from substrate-derived oxidative compounds toward fermentation-derived metabolites. Linear aldehydes associated with lipid oxidation declined rapidly in cereal substrates but persisted longer in oilseed matrices. Mid-fermentation in wheat and rye was associated with transient enrichment of alcohols, esters, and phenolic compounds, corresponding to increased aroma complexity before shifting toward amino acid-derived metabolites at later stages. Oilseed substrates retained branched and lipid-derived aldehydes longer, maintaining sharper and more pungent aroma characteristics. Rapeseed fermentation additionally generated nitriles and isothiocyanates from glucosinolate degradation. High-moisture conditions promoted formation of redox-related overflow metabolites, including acetic acid and vicinal diketones.

**Discussion:**

The results demonstrate that aroma development during *A. oryzae* SSF is governed by a substrate- and moisture-dependent metabolic continuum, where evolving water activity and enzyme-mediated substrate conversion dynamically shape metabolic routing and volatilome composition. These findings highlight the importance of fermentation stage, substrate chemistry, and moisture dynamics in controlling aroma formation during koji fermentation of diverse agro-industrial side-streams.

## 1 Introduction

The food system alone accounts for one-third of the total greenhouse gas emissions (GHG) worldwide (Crippa et al., 2021), and from the total human-caused GHG emissions, about 8–10 % are reported to come from food waste (IPCC, 2019). Reducing the current extensive level of food waste is essential to establish resource-efficient and sustainable food systems. The use of a new concept is increasing in the spectrum of food waste reduction, reporting a huge market potential by tendency reports, and this is upcycled food (Ewing-Chow, 2021; Zaraska, 2021). Meaningfully, the economic worth of agro-industrial waste can be readily enhanced through fungal pretreatment within solid-state fermentation (SSF) systems. By-products from the food industry are often characterized by valuable nutritional value and containing significant quantities of bioactive compounds (Herrero et al., 2015). SSF offers the potential to produce high-value-added products by utilizing these residues as substrates (El Sheikha and Ray, 2023).

Solid-state fermentation (SSF) is a well-established biotechnological process in which microorganisms grow on moist solid substrates in the absence of free water, enabling the efficient conversion of complex raw materials into value-added products. Owing to its low water and energy requirements, high product yields, and suitability for enzyme and flavor production, SSF has gained increasing attention as an environmentally sustainable processing strategy, particularly for the valorization of agricultural by-products (Ravindran and Jaiswal, 2016).

Among SSF systems, koji fermentation represents a traditional and industrially relevant application, relying predominantly on filamentous fungi of the species *Aspergillus oryzae*. Koji serves as a key intermediate in the production of fermented foods such as soy sauce, miso, and rice wine, where fungal enzymes hydrolyze starches and proteins into fermentable sugars, amino acids, and flavor precursors. In addition to its enzymatic activity, koji fermentation is a major contributor to aroma formation, generating a wide range of volatile organic compounds (VOCs) that strongly influence the sensory quality of fermented foods (Chou and Ling, 1998; Gao et al., 2010).

Fermentation outcomes in SSF are strongly shaped by substrate composition and process parameters, including moisture content, temperature, oxygen availability, and fermentation time. These factors regulate microbial growth, metabolic activity, and the balance between carbohydrate and protein degradation, which can be monitored through physicochemical indicators such as pH, water activity, reducing sugars, and primary amines (Li et al., 2023; Moirangthem et al., 2024; Lux et al., 2026; Kabir et al., 2025; Martău et al., 2021). Moreover, variations in these parameters have been shown to affect VOC profiles and aroma characteristics, yet the relationships between substrate properties, fermentation conditions, and aroma development remain insufficiently characterized.

This study investigates solid-state koji fermentation using *Aspergillus oryzae* on cereal- and oilseed-derived substrates, focusing on fungal growth, physicochemical changes, metabolic activity, and aroma formation. While koji fermentation has been extensively studied in rice- and soybean-based systems, comparatively little is known about the fermentation behavior of cereal and oilseed side-streams, and moisture is often treated as a fixed initial parameter rather than a dynamic process variable. By linking substrate-specific properties with fermentation performance and volatile profiles, this work aims to improve the understanding of how SSF parameters shape metabolic and sensory outcomes, contributing to the targeted design of fermented ingredients from food processing by-products.

## 2 Materials and methods

### 2.1 Chemicals

SDS (10% w/w), borate (0.1 M), Na-MES (200 g/L), OPA (40 g/L in ethanol), perchloric acid (0.5 M), Triton X −100 (100 g/L), L-glutamic acid stock (100 mmol/L), 3,5-dinitrosalicylic acid (DNS), D-(+)-Glucose (2 mM).

### 2.2 Materials

Wheat and rye bran were obtained from the production of wheat and rye flour, respectively, at Skærtoft Mølle (Denmark). Commercial warm-pressed rapeseed press cake was sourced from Scanola Oil Factory (Denmark). The materials were delivered to the university in June 2022, vacuum-packed in 5 kg bags, and stored at 5 °C until use. The residual lipid content of the rapeseed press cake was 9.7%. Peeled pumpkin seeds were purchased from DK-NUTS ApS and roasted at 170 °C for 10 min prior to oil extraction. Oil pressing was performed using an OW100 s-inox press (Ölwerk). The press was preheated to 150 °C for 10 min, set to set to the manufacturer-defined calibration setting (“one turn”), and equipped with a 10 mm pressing nozzle. From 5 kg of pumpkin seeds, 1.020 kg of oil and 3.784 kg of pumpkin seed press cake were obtained. *Aspergillus oryzae* (White Koji) spores were purchased from fermentationculture.eu (LUVI Fermente KG).

### 2.3 Methods

#### 2.3.1 Koji fermentation

Duplicate fermentations (*n* = 2) were organized into batches in which all experiments shared identical baseline conditions, with only a single variable modified per batch. Initial *added water* (expressed as %(w/w) of total substrate mass) was selected as the first parameter investigated, serving as a preliminary step to establish the default moisture level. Grain substrates exhibited comparable behavior; therefore, identical added water levels (60% and 70%) were applied across grain experiments. In contrast, oil-cake substrates required distinct added water settings (20%, 30% and 40%). Fermentation duration varied to evaluate shorter incubation periods (30, 40, and 48 h).

The selected added water levels were based on typical moisture ranges reported for solid-state fermentation and adjusted according to substrate characteristics. SSF systems generally operate within a moisture range of approximately 30–70%, depending on substrate water-holding capacity and the minimum water activity required for fungal growth (Pandey, 2003; Mitchell et al., 2006). Cereal substrates such as wheat and rye bran exhibit high water absorption capacity and structural porosity, supporting higher moisture levels (60–70% w/w), whereas oilseed press cakes are denser and contain residual lipids, resulting in lower optimal moisture ranges (20–40% w/w) to maintain suitable aeration and avoid excessive compaction.

Koji fermentations were performed in batches under identical experimental conditions. For each batch, 200 g of substrate was mixed with tap water to achieve the designated added water content. The hydrated substrates were steamed in perforated baking trays covered with kitchen towels using a Rational D-86899 oven (RATIONAL) at 100 °C for 10 min. After steaming, substrates were transferred to plastic containers and cooled at room temperature to below 25 °C. From each sample, 50 g was collected as an unfermented control prior to inoculation, while the remaining 150 g was inoculated with 2.4 milligrams of *Aspergillus oryzae* spores at the dosage recommended by the supplier (0.016%) and thoroughly mixed to ensure uniform spore distribution.

The inoculated substrates were returned to perforated baking trays covered with moistened kitchen towels and incubated without ventilation at 30 °C and 70% relative humidity. Incubation was carried out using either a Termaks KB8182 cooling incubator (Nordic Labtech) or a Forma™ Steri-Cult™ CO_2_ incubator (Thermo Fisher Scientific). After 24 h, samples were manually mixed to promote homogeneous fungal growth. Fermentation was terminated at the designated time point, after which samples were removed from the incubator and stored at −25 °C until further analysis.

#### 2.3.2 Visual assessment of mycelial growth

Visual assessment of mycelial growth was performed by two assessors immediately after removal of the samples from the incubator. Mycelial coverage was scored using a semi-quantitative scale ranging from + (no or almost no visible mycelium) to +++ (extensive visible mycelium throughout the sample).

#### 2.3.3 Physiochemical and metabolic analysis

Physicochemical and metabolic analyses were performed to characterize changes during fermentation. Water content, water activity and pH were measured for all samples. Reducing sugars and primary amines were analyzed for all endpoint samples and for one representative unfermented control batch per substrate. Samples were prepared using a mortar to homogenize them. Then 1 g of the sample was added to 11 mL of distilled water. The solution was mixed using a vortex MS2 Minishaker (IKA^®^-Werke, Germany) for 10 seconds, then a tube revolver SB3 Rotator (Stuart^®^, Great Britain) for 30 minutes, and centrifuged in a refrigerated benchtop centrifuge Sigma 4–16KS (Sigma Laborzentrifugen, Germany) for 5 min at 2,100 RCF and 4 °C. The obtained supernatant was transferred to a fresh falcon tube and kept frozen at −25 °C until usage.

#### 2.3.4 Water content and water activity

Water content was determined using an MA 30 infrared moisture analyzer (Sartorius) by drying approximately 3.0 g of sample at 105 °C until constant weight. Water activity was measured at 25 °C using an Aqualab PRE analyzer (METER Group). For pH determination, 1 g of sample was diluted in 4 mL of distilled water, vortexed, equilibrated for 15 min, and measured in triplicate using an edge^®^ pH meter (HannaNorden). Soluble solids were measured using a digital refractometer (HI-96800; Hanna Instruments) on the same dilutions used for pH measurement.

#### 2.3.5 Determination of reducing sugars

The 3,5-dinitrosalicylic acid (DNS) reagent was prepared by first dissolving 4.38 g DNS in 200 mL Milli-Q water at 60 °C with stirring (solution 1). Solution 2 was prepared by mixing 40 mL of 4 M NaOH with 40 mL Milli-Q water, followed by the addition of 120 g sodium potassium tartrate and 120 mL of Milli-Q water at 40 °C. Solutions 1 and 2 were combined to obtain the final DNS reagent (0.1% DNS, 30% sodium potassium tartrate, 0.4 M NaOH). A standard of glucose (0–1.0 mmol/L) was prepared in Milli-Q water. For the preparation of standards, 1 mL of DNS reagent was added to each test tube containing the appropriate standard solution and incubated in boiling water for 5 min. The tubes were subsequently cooled to room temperature in an ice-cold bath. Sample preparation consisted of mixing 0.2 mL of sample supernatant with 1 mL DNS reagent and 0.8 mL Milli-Q water, followed by vortexing. Samples were treated identically to the standards, with 5 min boiling and cooling in an ice-cold water bath. For spectrophotometric measurement, 300 μL of each standard or sample was dispensed in triplicate (100 μL per well) on a 96-well microplate. Absorbance was measured at 540 nm using a BioTek Epoch 2 Microplate Spectrophotometer (Agilent Technologies, USA). Glucose was used to generate calibration curves for estimating reducing sugar equivalents in the samples, with a linear fit and *R*^2^ of 0.98, y = 1.66 x −0.01.

#### 2.3.6 Determination of primary amines by OPA assay

Primary amines were quantified using the o-phthaldialdehyde (OPA) method. OPA working solution (50 mL) was prepared fresh by combining 25 mL borate solution (0.1 M), 5 mL SDS solution (10% w/w), 1 mL OPA solution (40 g/L in ethanol), 1 mL 2-mercapto-ethansulfonic acid solution (Na-MES, 200 g/L), 2.5 mL Triton X-100 solution (100 g/L), and distilled water up to 50 mL. The borate solution was prepared freshly for each experiment, while other reagents were stored at −18 °C until use. L-glutamic acid standards were prepared in serial dilutions (0–8.0 mmol/L) using 0.5 M perchloric acid as diluent. For the assay, 232 μL of OPA working solution was mixed with 8 μL of sample or standard in a 96-well microplate, mixed, and incubated for 10 min at 30 °C in the dark. Absorbance was measured at 335 nm using a microplate reader (same as for the DNS assay). Sample primary amine content was calculated from the L-glutamic acid standard curve, for estimating primary amine equivalents in the samples, with a linear fit and *R*^2^ of 1.00, y = 0.22 x + 0.12.

#### 2.3.7 Dynamic head-space GC-MS

Aroma profiles were analyzed by GC–MS using dynamic headspace sampling with Tenax traps (200 mg Tenax TA 60/80—Mesh, Perkin Elmer). One measurement was performed per fermentation condition (substrate × moisture × time). One replicate from pumpkin (40% initial water, 48 h) was excluded due to technical variability caused by residual moisture in the Tenax trap, which elevated the chromatographic baseline and prevented reliable quantification. In total, 37 samples were included in the final analysis). Samples were homogenized with an UltraTurrax mixer at 13,000 rpm for 90 seconds and purged for 40 min with 100 ml/min pure nitrogen at 37 °C with 200 rpm magnetic stirring. Dry purging (100 mL/min) with pure nitrogen was performed for 10 min. Primary desorption was carried out by heating the trap to 250 °C with a flow (50 mL/min) of carrier gas for 15 min. The tripped volatiles were trapped in a secondary Tenax-TA cold trap (30 mg held at 1 °C), which was subsequently heated at 280 °C for 4 minutes (secondary desorption, outlet split 1:10). This allowed for rapid transfer of volatiles to a gas chromatograph-mass spectrometer (GC-MS, 7890A GC-system interfaced with a 5975C VL MSD with Triple-Axis detector from Agilent Technologies, USA) through a heated (225 °C) transfer line. Separation of volatiles was carried out on a ZB-Wax capillary column 30 m long x 0.25 mm internal diameter, 0.50 μm film thickness. The column pressure was held constant at 2.3 psi resulting in an initial flow rate of 1.4 mL/min using hydrogen as carrier gas. The column temperature program was: 10 min at 35 °C, from 35 °C to 240 °C at 8 °C min−1, and finally 5 min at 240 °C. The mass spectrometer was operating in the electron ionization mode at 70 eV. Mass-to-charge ratios between 15 and 300 were scanned.

#### 2.3.8 Data analysis

Untargeted profiling of GC–MS raw data was performed using PARADISe software (Version 6.1.7; https://ucphchemometrics.com/paradise/) Flexible coupling PARAFAC2 models with non-negativity constraints in all modes were fitted for each chromatographic interval. Relative compound abundances (peak areas), together with resolved mass spectra, were exported into a peak table. Compound identification was based on spectral similarity against the NIST11 MS database (NIST/EPA/NIH Mass Spectral Library, NIST Scientific and Technical Databases, Gaithersburg, MD, USA) using MS Search 2.0 implemented in PARADISe. Compounds with a match factor < 700 were excluded. Retention times were converted to linear retention index (LRI) values for additional validation, calculated using a homologous series of n-alkanes (C6–C22; Hewlett-Packard Co., Avondale, PA, USA). Compounds with a deviation of >±50 from reported values at the Volatile Compounds in Food (VCF; https://www.vcf-online.nl/) database or PubChem (https://pubchem.ncbi.nlm.nih.gov) were excluded. The final list of 53 volatile compounds meeting the criteria mentioned above can be found in supplementary data 2, including resolved mass spectra, NIST search results, peak areas, retention index, and odor descriptors.

Statistical analyses were conducted in R version 4.4.1 (R Foundation for Statistical Computing, Vienna, Austria). Peak areas from validated compounds were log_2_-transformed and scaled prior to heatmap generation. Heatmaps were produced using the *ComplexHeatmap* package (v. 2.20.0), and hierarchical clustering was performed using the *hclust* function with Euclidean distance and Ward's method (Ward.D).

## 3 Results

### 3.1 Mycelial growth was affected by initial added water

**Figure 1:**
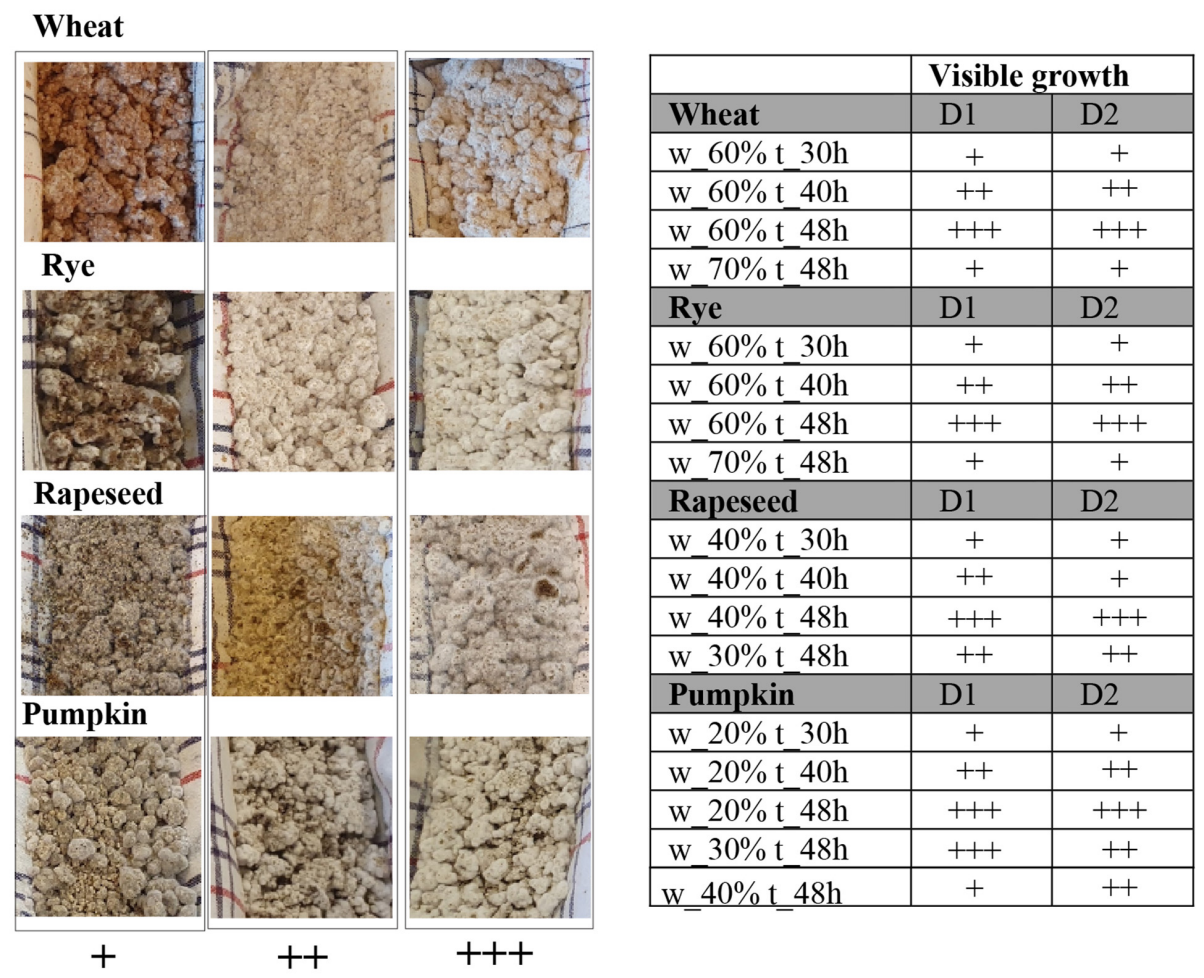
Variations in visible mycelial growth between duplicate fermentations (*n* = 2; D1 and D2) of different substrates following fermentation with *Aspergillus oryzae*, resulting from differences in initial added water content or fermentation duration, are summarized in the growth scoring table and illustrated by representative images. Samples differed only in the parameters indicated. Growth scoring was based on visual assessment: + indicates minimal visible growth with little to no detectable mycelium and disintegration of the koji structure; ++ indicates moderate surface mycelial growth without full penetration of the substrate and partial structural cohesion; +++ indicates extensive and homogeneous mycelial growth across the surface and throughout the substrate, with well-developed mycelium and intact structural integrity.

Visible mycelial growth was used as a qualitative indicator of fermentation performance and was assessed immediately after removal of the samples from the incubator. Wheat and rye substrates inoculated with *Aspergillus oryzae* exhibited comparable levels of visible mycelial development, whereas pumpkin consistently showed the lowest degree of surface and internal mycelial growth across all tested conditions (Figure 1). For rapeseed press cake, enhanced mycelial development was observed at an initial added water content of 40%. Some variability between biological duplicates was observed for pumpkin fermented at 30% and 40% initial added water, although overall growth remained lower compared to the cereal substrates.

### 3.2 Substrate-dependent changes in physicochemical and metabolic profiles

**Figure 2:**
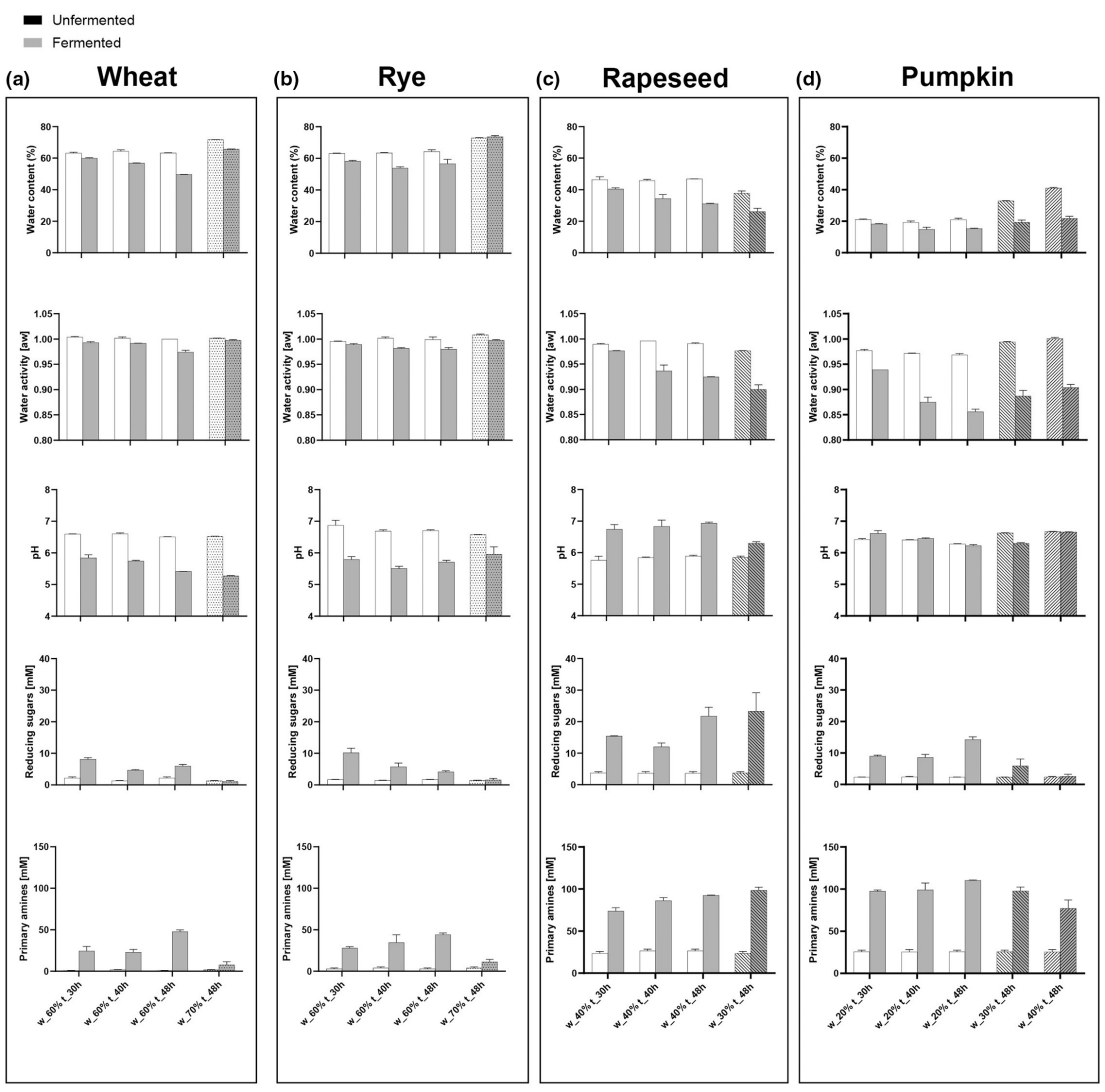
Physicochemical properties of pH, water activity, and water content and metabolite concentrations of reducing sugars and primary amines of four substrates: **(a)** Wheat bran; **(b)** Rye bran; **(c)** Rapeseed press; and **(d)** Pumpkin press cake, inoculated with *Aspergillus oryzae*, measured before and after fermentation. The data visualized is averages and range between duplicates fermentations (*n* = 2). The figure was generated using GraphPad PRISM version 10.

Across all substrates, fermentation resulted in measurable shifts in pH, water content, water activity, reducing sugar concentrations, and primary amine levels, although the magnitude and direction of these changes differed by substrate (Figure 2).

For wheat and rye bran, fermentation led to decreases in pH and reductions in both water content and water activity, accompanied by pronounced increases in reducing sugars and primary amines (Figure 2a, b). The physicochemical and metabolic profiles of wheat and rye were broadly similar. Declining moisture and water activity during *A. oryzae* SSF have previously been reported for cereal substrates, reflecting progressive changes in water distribution and binding within the fermenting matrix (Bechman et al., 2012; Jin et al., 2019).

Rapeseed press cake exhibited increases in pH together with pronounced increases in reducing sugars and primary amines, while water content and water activity decreased slightly during fermentation (Figure 2c). Comparable moisture declines during *Aspergillus*-mediated SSF have been associated with ongoing hydrolysis and structural changes in the substrate matrix (Jin et al., 2019).

Pumpkin press cake showed minimal changes in pH but substantial decreases in water activity. Primary amine levels increased notably, whereas increases in reducing sugars were comparatively limited (Figure 2d). Variability in hydration behavior during SSF has previously been linked to substrate composition and water-binding capacity (Jin et al., 2019).

Overall, the results Figure 2 demonstrates that SSF with *A. oryzae* induces substrate-dependent physicochemical and metabolic changes. Bran-based substrates showed concurrent increases in reducing sugars and primary amines, whereas oilseed-derived substrates exhibited relatively stronger increases in primary amines compared with reducing sugars. These trends are consistent with the observed mycelial growth patterns, where higher growth scores correspond to more pronounced physicochemical changes, reflecting increased enzymatic activity and substrate turnover.

The physicochemical changes reflect substrate-dependent metabolic routing during fermentation. In rapeseed press cake, the increase in pH and primary amines suggests enhanced proteolysis and possible ammonia formation from nitrogen metabolism, consistent with its high protein content. In contrast, wheat and rye showed acidification alongside increases in sugars and amines, indicating more balanced carbohydrate and protein degradation. These differences show that substrate composition directly influences the observed physicochemical trajectories.

### 3.3 Effects of fermentation conditions on koji aroma profiles

#### 3.3.1 Principle component analysis of replicates

**Figure 3:**
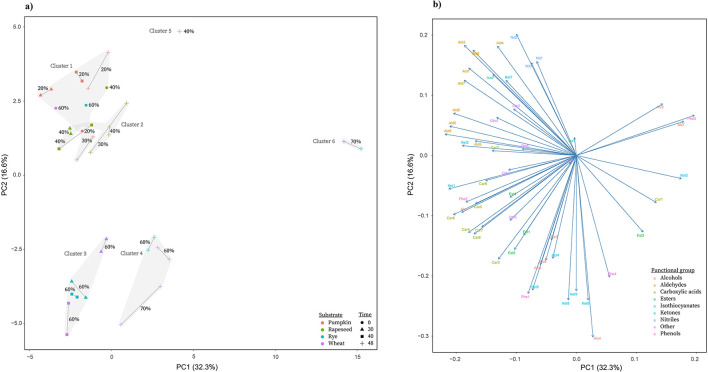
Principal component analysis (PCA) of volatile profiles obtained by GC–MS during solid-state fermentation with Aspergillus oryzae. **(a)** Score plot showing sample distribution based on log_2_-transformed and scaled relative abundances of volatile compounds. Points represent individual fermentation conditions, colored by substrate and shaped by fermentation time. Clusters (1–6) indicate unsupervised grouping based on hierarchical clustering (Euclidean distance, Ward's method). Grey polygons indicate cluster groupings. **(b)** Loading plot showing the contribution of individual volatile compounds to PC1 and PC2. Compounds are colored by functional groups (alcohols, aldehydes, carboxylic acids, esters, isothiocyanates, ketones, nitriles, phenols, and others). See supplementary data for list of compounds and abbreviations.

Principal component analysis (PCA) was performed to provide an overview of the global structure of the volatilome data and to assess the consistency between duplicate fermentations (Figure 3a). The first two principal components explained a substantial proportion of the total variance (PC1: 32.3%, PC2: 16.6%), capturing the dominant patterns associated with substrate type and fermentation progression. Replicate fermentations generally clustered in close proximity in the PCA space, indicating overall reproducibility of the GC–MS measurements, although some variability between duplicates was observed, particularly for pumpkin and high-moisture conditions, where replicate pairs occasionally showed noticeable separation (Figure 3a).

At the global level, samples separated primarily according to substrate along PC1, with cereal-based substrates (wheat and rye) forming a distinct group from oilseed-derived substrates (rapeseed and pumpkin), while PC2 captured a temporal trajectory, particularly for wheat and rye, where samples progressed from early (30 h) to mid (40 h) and late (48 h) fermentation stages. Inspection of the loading plot (Figure 3b) shows that this separation is driven by distinct compound classes, with unfermented samples and oilseed substrates associated with aldehydes, consistent with substrate-derived lipid oxidation products, whereas wheat and rye fermentations were associated with ketones, esters, and alcohols, reflecting the emergence of fermentation-derived volatiles.

#### 3.3.2 Phenotypic clustering of samples

**Figure 4:**
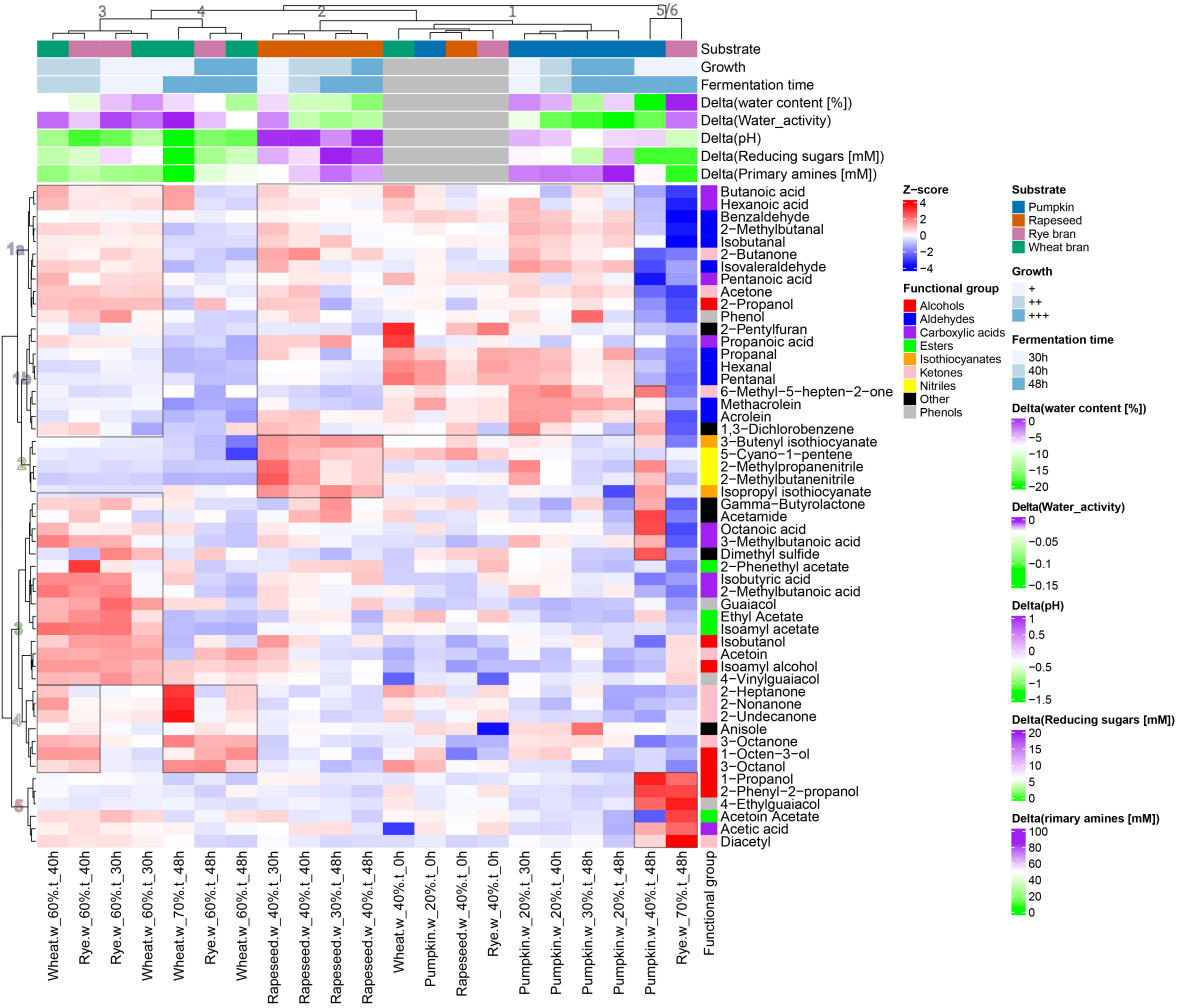
Hierarchical clustering heatmap of volatile compounds and physicochemical changes during solid-state fermentation with *Aspergillus oryzae*. Columns represent individual samples across substrates (wheat bran, rye bran, rapeseed press cake, and pumpkin press cake), fermentation times (30, 40, and 48 h), and initial added water contents, with growth scoring indicated above each column. Rows show log_2_-transformed, z-score–scaled relative average abundances of volatile compounds from duplicate fermentations (*n* = 2), grouped by functional class (alcohols, aldehydes, carboxylic acids, esters, isothiocyanates, ketones, nitriles, other compounds, and phenols). Upper annotation bars display substrate type, growth score, fermentation time, and changes (Δ) in water content, water activity, pH, reducing sugars, and primary amines. Hierarchical clustering was performed using Euclidean distance and Ward's method (Ward.D2). Numbers above the dendrogram (1–6) denote major sample clusters, also shown in Figure 3, reflecting distinct fermentation states across substrates, time, and moisture conditions. In contrast, row-wise clustering resolves compound clusters (1a, 1b, 2–5) corresponding to distinct metabolic regimes. Black boxes highlight the trends in clusters of compounds with similar abundance patterns: (1a) short-chain carboxylic acids and strecker aldehydes (1b) linear aldehydes and related lipid oxidation products; (2) nitrogen- and sulfur-containing compounds including nitriles and isothiocyanates together with acetamide, γ-butyrolactone, and propanoic acid; (3) alcohols, esters, phenols, aldehydes, ketones, and; (4) methyl ketones and fungal lipid-derived compounds including 1-octen-3-ol; and (5) acetic acid, diacetyl, acetoin acetate, 2-phenyl-2-propanol, 4-ethylguaiacol, and 1-propanol.

Untargeted GC–MS analysis revealed pronounced substrate- and condition-dependent variation in volatile organic compounds (VOCs) (Figure 4). The detected compounds included alcohols (e.g., ethanol, 2-propanol, 1-propanol, isoamyl alcohol, and 1-octen-3-ol), aldehydes (e.g., isovaleraldehyde, benzaldehyde, hexanal, and pentanal), ketones (e.g., acetoin, diacetyl, 2-heptanone, 2-nonanone, and 2-undecanone), carboxylic acids (e.g., acetic acid, isobutyric acid, 3-methylbutanoic acid, and 2-methylbutanoic acid), esters (e.g., ethyl acetate and isoamyl acetate), phenolic compounds (e.g., guaiacol, 4-vinylguaiacol, and 4-ethylguaiacol), and glucosinolate-derived sulfur compounds including isothiocyanates and nitriles.

Hierarchical clustering of the volatile heatmap (Figure 4) resolved five major compound clusters, reflecting distinct aroma axes shaped by substrate composition, fermentation stage, and moisture conditions. Across clusters, a consistent pattern emerges where cereal substrates show a transition toward fermentation-derived volatiles at 30–40 h, whereas oilseed substrates, particularly at lower added water levels, retain stronger contributions from substrate-derived compounds throughout fermentation.

#### 3.3.3 Cluster 1—linear aldehydes and substrate-derived lipid oxidation products

Cluster (1) comprises linear aldehydes and related lipid oxidation products including propanal, pentanal, hexanal, acrolein, methacrolein, and 2-pentylfuran (Figure 4). These compounds were present at the highest relative levels in the untreated substrates prior to fermentation. During fermentation, linear aldehydes rapidly declined and were no longer detectable in wheat and rye after 30 h. In contrast, they persisted in pumpkin press cake throughout fermentation and, to a lesser extent, in rapeseed press cake, where a progressive decrease was observed over time. Acrolein and methacrolein were not detected after fermentation. Overall, this cluster displayed a clear substrate-dependent pattern, with rapid disappearance in cereal substrates and continued, though declining, presence in oilseed matrices (Figure 4).

#### 3.3.4 Cluster 2—nitrogen- and sulfur-containing compounds associated with rapeseed substrates

Cluster (2) comprised nitrogen- and sulfur-containing metabolites including acetamide, γ-butyrolactone, propanoic acid, isopropyl isothiocyanate, 2-methylbutanenitrile, 2-methylpropanenitrile, 3-butenyl isothiocyanate, and 5-cyano-1-pentene. Several of these compounds correspond to nitriles and isothiocyanates associated with glucosinolate degradation, reflecting the Brassica origin of the rapeseed substrate. This cluster therefore represents a substrate-specific volatile signature linked to glucosinolate-derived chemistry.

#### 3.3.5 Cluster 3—fermentation-derived alcohols, esters and amino acid degradation products

Cluster (3) represented the dominant fermentation-associated volatile regime and was characterized by alcohols, esters, phenolic compounds, aldehydes, ketones, and short-chain carboxylic acids. Compounds contributing to this cluster included higher alcohols (e.g., isoamyl alcohol and isobutanol), fermentation metabolites such as acetoin and acetone, ethanol-derived esters (e.g., ethyl acetate, isoamyl acetate, and 2-phenethyl acetate), phenolic compounds (e.g., guaiacol and 4-vinylguaiacol), as well as branched aldehydes and acids including isovaleraldehyde, 2-methylbutanal, and 3-methylbutanoic acid.

Wheat and rye clustered together and showed a pronounced enrichment of these compounds at 30 h and 40 h. This enrichment was transient and declined at 48 h (Figure 4). In contrast, rapeseed and pumpkin did not show the same coordinated mid-fermentation increase. In these substrates, the relative abundance of fermentation-associated alcohols and esters was generally lower and more dispersed across time points. Overall, the fermentation-derived cluster was most clearly defined and temporally concentrated in wheat and rye at 30–40 h, whereas oilseed substrates displayed a weaker and less synchronized pattern.

#### 3.3.6 Cluster 4—fungal lipid metabolism and methyl ketones

Cluster (4) consisted primarily of methyl ketones and fungal lipid-derived compounds including 2-heptanone, 2-nonanone, 2-undecanone, 3-octanone, 3-octanol, and the characteristic mushroom alcohol 1-octen-3-ol (Figure 4). These compounds were present at very low levels or absent in the untreated substrates but increased during fermentation. Their relative abundance generally increased with fermentation time, with the highest levels observed at 48 h.

Substrate-specific patterns were evident within this cluster. Wheat bran samples showed the strongest enrichment, particularly for 2-heptanone, 2-nonanone, 2-undecanone, and 3-octanol. Pumpkin press cake also showed increased levels of 2-heptanone and 3-octanol, whereas rye bran displayed lower and more scattered abundances of these compounds.

Notably, the highest levels within this cluster were observed in wheat fermented for 48 h at 70% added water, despite reduced visible mycelial growth under these conditions (Figure 4). This indicates that formation of these lipid-derived volatiles can remain pronounced even when biomass expansion is limited.

#### 3.3.7 Cluster 5—high-moisture deviation cluster

Cluster (5) was characterized by acetic acid, diacetyl, acetoin acetate, 2-phenyl-2-propanol, 4-ethylguaiacol, and 1-propanol. A distinct grouping of samples consisting of pumpkin fermented at 40% water added and rye fermented at 70% water added showed elevated levels of these compounds. The accumulation of acetic acid and vicinal diketones suggests altered redox balance and partial fermentative overflow under high-moisture conditions.

## 4 Discussion

This study provides one of the first GC–MS–resolved characterizations of volatile formation during *Aspergillus oryzae* koji fermentation of cereal brans and oilseed press-cake side-streams, extending previous fermentation and metabolite evolution studies in solid-state systems (Couto and Sanromán, 2006; Gao et al., 2010; Cooray and Chen, 2018; El Sheikha and Ray, 2023; Hwang and Kim, 2023; Yu et al., 2023; Šelo et al., 2024; Kim et al., 2025). While koji volatilome research has traditionally focused on rice and soybean systems, and fermentation of oilseed press cakes has largely emphasized nutritional upgrading, the present work demonstrates how substrate composition, enzyme-driven substrate conversion, and dynamic moisture evolution jointly shape metabolic and aroma outcomes in underexplored agro-industrial side-streams. By integrating growth assessment, water activity changes, pH development, primary amine accumulation, reducing sugar formation, and volatilome profiling, this study establishes a condition-resolved mapping of fermentation behavior across chemically distinct substrates. The clustering patterns observed in Figure 4 highlight that fermentation outcomes are not governed by initial added water levels alone, but by their interaction with substrate properties and fermentation progression. Samples do not group according to initial moisture conditions in isolation; rather, distinct trajectories emerge for each substrate, with additional separation under high-moisture conditions. This indicates that moisture acts as a dynamic modulator of fermentation behavior, where changes in water content and water activity during fermentation contribute to shaping metabolic routing and aroma development.

SSF is governed by interacting gradients in water availability, oxygen diffusion, and matrix structure, which regulate fungal growth, enzyme secretion, and metabolite formation (Mitchell et al., 2006; Lee et al., 2016). Classical SSF studies have shown that water content and water activity decline progressively during *A. oryzae* fermentation due to redistribution of water and increasing binding within the substrate matrix (Bechman et al., 2012; Jin et al., 2019). Consistent with these findings, water activity decreased across all substrates in the present study, although the magnitude differed substantially. Rapeseed and pumpkin experienced stronger moisture and water activity loss than cereal substrates, likely reflecting differences in matrix composition, water-binding capacity, and porosity. Jin et al. (2019) demonstrated that water becomes increasingly bound during fermentation, accompanied by structural and physicochemical changes in the substrate, supporting the observed dehydration patterns in oilseed press cakes.

Growth behavior and metabolic indicators were consistent with established relationships between enzyme production, substrate hydrolysis, and environmental conditions in koji fermentation. Studies of rice and soybean koji have shown that secretion of key hydrolytic enzymes, including α-amylase, glucoamylase, and acidic protease, is strongly influenced by moisture level, substrate composition, and fermentation time (Chancharoonpong et al., 2012; Lin et al., 2014). In the present study, wheat and rye bran fermentations followed similar trajectories characterized by decreasing pH and concurrent increases in reducing sugars and primary amines, consistent with coordinated carbohydrate and protein hydrolysis mediated by these enzyme systems. In contrast, rapeseed press cake exhibited pronounced alkalinization despite comparable primary amine accumulation. Because the OPA assay quantifies primary amines but does not detect ammonia, nitrogen mineralization via amino acid deamination may be underestimated (Nielsen et al., 2001). The pH increase observed during rapeseed press cake fermentation is therefore most consistent with dominant nitrogen catabolism relative to organic acid accumulation.

The volatilome patterns observed in Figure 4 can be interpreted through five compound clusters reflecting distinct metabolic regimes.

The first regime (Cluster 1) represents substrate-derived lipid oxidation products; most pronounced in rapeseed and partly in pumpkin substrates. Linear aldehydes such as propanal, pentanal, and hexanal originate from oxidative degradation of unsaturated fatty acids and were present in the untreated substrates together with thermal degradation markers such as acrolein and methacrolein. During fermentation these compounds declined, disappearing rapidly in wheat and rye while persisting longer in pumpkin and, to a lesser extent, rapeseed. This pattern suggests that cereal fermentations more rapidly transition away from substrate-derived oxidative chemistry, whereas oilseed matrices retain lipid-derived oxidation products longer.

A second cluster (Cluster 2) comprised nitrogen- and sulfur-containing compounds associated with rapeseed substrates, including nitriles and isothiocyanates derived from glucosinolate degradation. Rapeseed press cake originates from Brassica seeds rich in glucosinolates, which are enzymatically converted into nitriles and isothiocyanates during processing and microbial transformation. GC–MS analysis revealed compounds such as isopropyl isothiocyanate and 3-butenyl isothiocyanate, which are known products of glucosinolate breakdown (Shi et al., 2015). These sulfur-containing volatiles are associated with bitter, pungent, mustard-like, and sulfurous sensory characteristics (Bell et al., 2018; Marcinkowska and Jeleń, 2020; Bell et al., 2021) and represent an important substrate-specific dimension of flavor development in fermented *Brassica* side-streams.

Cluster 3 corresponded to fermentation-derived alcohols, esters, phenols, and amino acid degradation products, most clearly expressed in wheat and rye bran fermentations. These compounds arise from carbohydrate metabolism and amino acid catabolism and were enriched during mid-fermentation (30–40 h), when fungal colonization and enzymatic activity were most pronounced. The shift in the volatome could either reflect increased oxygen availability and evaporation over the last 8 h of the fermentation or fungal enzymatic activity. Notably, the observed decline in acetate esters at 48 h compared with earlier stages is consistent with increasing esterase activity during later stages of fungal growth. Koji molds possess substantial esterase capacity (Higuchi et al., 1991), and genome analysis of *A. oryzae* confirms the presence of multiple genes encoding secreted esterases and related hydrolases (Machida et al., 2005). Time-resolved secretome analyses in filamentous *Aspergillus* species have further demonstrated that extracellular hydrolases, including esterase- and lipase-class enzymes, are differentially expressed during solid-state cultivation and become enriched under nutrient limitation and stationary-phase–associated conditions (Saykhedkar et al., 2012). Together, these observations provide a plausible explanation for the reduced abundance of isoamyl acetate, phenethyl acetate, and ethyl acetate at 48 h as fermentation progresses.

Cluster 4 reflected fungal lipid metabolism, characterized by the appearance of methyl ketones and oxylipin-related compounds including 1-octen-3-ol. The mushroom alcohol 1-octen-3-ol is a character impact compound for koji aroma (Feng et al., 2013). High levels of methyl-ketones were uniquely associated with wheat bran fermentations, increasing at 60% and reaching their highest levels at 70% water added despite reduced visible growth. The occurrence of 2-heptanone, 2-nonanone and 2-undecanone suggests formation via β-oxidative shortening of medium-chain fatty acids (C8–C12) or intermediates derived from longer lipids (Forney and Markovetz, 1971). Such methyl-ketone series are consistent with progressive lipid turnover and chain-shortening during fungal metabolism. This indicates that volatile formation can become partially decoupled from biomass expansion and reflects altered metabolic routing under high-moisture conditions, where internal oxygen gradients and matrix restructuring are known to influence fungal metabolism in solid-state fermentation systems.

Finally, Cluster 5 represented a high-moisture deviation cluster, comprising rye at 70% and pumpkin at 40% water added, was characterized by elevated acetic acid, diacetyl/acetoin, and 4-ethylguaiacol. Diacetyl and acetoin originate from pyruvate via the α-acetolactate pathway and are common fermentation metabolites (Xiao and Xu, 2007). In solid-state fermentation systems, increasing moisture reduces gas-filled porosity in the substrate matrix and restricts oxygen diffusion, potentially creating localized oxygen limitations within the matrix (Pandey, 2003; Mitchell et al., 2006). Under such conditions, metabolic flux may be diverted away from fully oxidative metabolism toward pyruvate overflow routes such as the α-acetolactate pathway, leading to accumulation of diacetyl and acetoin. The origin of these compounds cannot be unambiguously assigned to *A. oryzae*, as they may arise from fungal overflow metabolism, redox-balancing reactions, or minor contributions from indigenous microbiota under high-moisture conditions. The presence of 4-ethylguaiacol may reflect reduction of vinyl phenols derived from ferulic acid, a transformation commonly associated with yeasts rather than filamentous fungi (Tchobanov et al., 2008), although its precise origin in the present system cannot be resolved.

Collectively, fermentation reduced several substrate-derived off-flavor compounds, including lipid oxidation–associated aldehydes and 2-pentylfuran, indicating a transition away from raw oxidative products toward fermentation-derived volatiles. In rapeseed press cake, GC–MS analysis further revealed formation of glucosinolate-derived nitriles and isothiocyanates originating from enzymatic glucosinolate breakdown.

As the present study relies on relative GC–MS quantification without direct sensory analysis, the reported aroma descriptors reflect literature-based odor associations rather than measured sensory perception. However, the observed compound trajectories provide a basis for tentative interpretation of how the overall aroma profile may evolve during fermentation. The volatilome trajectories show a clear time-dependent evolution of aroma-relevant compounds. Early stages are dominated by linear aldehydes, characteristic products of lipid oxidation that are commonly described as green, fatty, and sharp in aroma. These compounds decline rapidly in cereal substrates during fermentation. Mid-fermentation in wheat and rye is marked by transient enrichment of alcohols, esters, and phenolic compounds, classes of volatiles often associated with floral, fruity, and lightly spicy aroma attributes and representing the greatest diversity of fermentation-derived volatiles during this phase. In contrast, oilseed substrates retain lipid-derived aldehydes longer and maintain branched aldehydes into later stages, compounds frequently described as sharp, earthy, or pungent. In rapeseed specifically, glucosinolate breakdown products including nitriles and isothiocyanates remain detectable throughout fermentation and are typically associated with bitter, mustard-like, and sulfurous aroma characteristics. Under high-moisture conditions, elevated levels of acetic acid and vicinal diketones were observed, compounds commonly described as sour, vinegary, or buttery in aroma.

While the present study establishes a framework linking substrate properties and moisture dynamics to volatile formation, several challenges remain for translation into food applications. The formation of glucosinolate-derived compounds in rapeseed, including nitriles and isothiocyanates, may contribute to undesirable bitter, pungent, or sulfurous notes, which could limit direct use without further process optimization or blending strategies. In addition, the structural properties of fermented side-streams, particularly oilseed press cakes, differ substantially from conventional food matrices, and texture development remains a key challenge for incorporation into finished products. From a regulatory perspective, the use of fermented side-streams and fungal biomass may also fall under novel food regulations in certain jurisdictions, requiring additional safety and documentation considerations.

Together, these results illustrate how substrate chemistry and moisture evolution define distinct metabolic aroma regimes during *A. oryzae* solid-state fermentation and highlight the potential of koji fermentation as a tool for flavor generation in food side streams.

## 5 Conclusion

This study demonstrates that aroma formation during Aspergillus oryzae solid-state fermentation is governed by substrate-dependent fermentation trajectories modulated by moisture dynamics rather than initial water addition alone. Cereal substrates (wheat and rye bran) exhibited coordinated physicochemical changes, including acidification and concurrent increases in reducing sugars and primary amines, accompanied by a transient enrichment of fermentation-derived alcohols, esters, and phenolic compounds. In contrast, oilseed press cakes showed substrate-specific responses, with rapeseed characterized by alkalinization, elevated amine formation, and accumulation of glucosinolate-derived nitriles and isothiocyanates, and pumpkin displaying less coordinated metabolic progression and greater variability.

Across conditions, volatilome clustering revealed a shift from substrate-derived lipid oxidation products toward fermentation-derived compounds, with distinct deviations under high-moisture conditions associated with increased levels of acetic acid, diacetyl, and related metabolites. These patterns indicate that moisture influences metabolic routing through changes in matrix structure and oxygen availability, thereby shaping the balance between oxidative and fermentative pathways.

These results show that fermentation time and appropriate substrate-dependent moisture levels are critical for the development of aroma profiles in solid-state fermentation.

## Data Availability

The original contributions presented in the study are included in the article/Supplementary material, further inquiries can be directed to the corresponding author/s.
